# Multifaceted Proteome Analysis at Solubility, Redox, and Expression Dimensions for Target Identification

**DOI:** 10.1002/advs.202401502

**Published:** 2024-08-09

**Authors:** Amir A. Saei, Albin Lundin, Hezheng Lyu, Hassan Gharibi, Huqiao Luo, Jaakko Teppo, Xuepei Zhang, Massimiliano Gaetani, Ákos Végvári, Rikard Holmdahl, Steven P. Gygi, Roman A. Zubarev

**Affiliations:** ^1^ Department of Cell Biology Harvard Medical School Boston MA 02115 USA; ^2^ Division of Chemistry I, Department of Medical Biochemistry and Biophysics Karolinska Institutet Stockholm SE‐17 177 Sweden; ^3^ Biozentrum University of Basel Basel 4056 Switzerland; ^4^ Department of Microbiology, Tumor and Cell Biology Karolinska Institutet Stockholm 17165 Sweden; ^5^ Division of Immunology, Medical Inflammation Research Group, Department of Medical Biochemistry and Biophysics Karolinska Institute Stockholm SE‐17 177 Sweden; ^6^ Drug Research Program, Faculty of Pharmacy University of Helsinki Helsinki FI‐00014 Finland; ^7^ SciLifeLab Stockholm SE‐17 177 Sweden

**Keywords:** auranofin, immunology, interferon, lupus, mass spectrometry, oxidation and reduction

## Abstract

Multifaceted interrogation of the proteome deepens the system‐wide understanding of biological systems; however, mapping the redox changes in the proteome has so far been significantly more challenging than expression and solubility/stability analyses. Here, the first high‐throughput redox proteomics approach integrated with expression analysis (REX) is devised and combined with the Proteome Integral Solubility Alteration (PISA) assay. The whole PISA‐REX experiment with up to four biological replicates can be multiplexed into a single tandem mass tag TMTpro set. For benchmarking this compact tool, HCT116 cells treated with auranofin are analyzed, showing great improvement compared with previous studies. PISA‐REX is then applied to study proteome remodeling upon stimulation of human monocytes by interferon *α* (IFN‐α). Applying this tool to study the proteome changes in plasmacytoid dendritic cells (pDCs) isolated from wild‐type versus *Ncf1*‐mutant mice treated with interferon *α*, shows that NCF1 deficiency enhances the STAT1 pathway and modulates the expression, solubility, and redox state of interferon‐induced proteins. Providing comprehensive multifaceted information on the proteome, the compact PISA‐REX has the potential to become an industry standard in proteomics and to open new windows into the biology of health and disease.

## Graphical abstract

Redox and EXpression (REX) is a high‐throughput and high‐depth proteomics approach that integrates the redox and expression facets. Combining REX with Proteome Integral Solubility Alteration (PISA) assay, PISA‐REX is implemented as an industry‐standard tool allowing for multifaceted proteomics profiling of a given biosystem at three dimensions of solubility, abundance, and redox state. PISA‐REX can triangulate the targets for drugs, biologicals, and diseases.

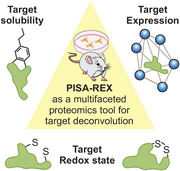

## 1 Introduction

Chemical proteomics employs tools for the interrogation of biological systems and their interactions with small molecules and other entities. Such tools can be used in target deconvolution and exploration of drug mechanisms of action by the analysis of changes in the protein abundance, solubility/stability, and redox state upon treatment with a biological or chemical entity.^[^ ^1^ ^]^ Such a multifaceted proteome interrogation can be extremely resource‐demanding. To alleviate this problem, we have recently developed a high‐throughput version of Thermal Proteome Profiling (TPP) or MS‐CETSA^[^ ^2^, ^3^ ^]^ called the Proteome Integral Solubility Alteration (PISA) assay.^[^ ^4^ ^]^ While in TPP, the samples heated to various temperature points are analyzed individually, in PISA assay, these samples are combined and analyzed as one sample using a single TMT channel. This allows for the inclusion of more replicates, contributing to the robustness of the downstream statistical analysis. The area under the curve is thus used as a proxy for calculating the solubility of the proteins upon a given perturbation. PISA drastically reduces the number of missing values, enhancing proteome coverage. By circumventing the need for sophisticated curve fitting algorithms, PISA assay is also less prone to false negative/positive discoveries. We combined PISA assay with expression proteomics (PISA‐Express) in the same tandem mass tag (TMT) multiplexing set to simultaneously track protein solubility/stability and expression changes upon cellular transitions to and from pluripotency.^[^ ^5^ ^]^ Multiplexing the whole experiment, including the desired number of biological replicates (≥3), into a single TMT set is highly beneficial, as it allows for robust normalization to protein abundance, and practically eliminates the problem of missing values.^[^ ^5^ ^]^ Due to its high throughput, PISA allows for more interactive experimental design, and we have used it to identify enzyme substrates,^[^ ^6^, ^7^ ^]^ to measure the residence time of drug binding on the target,^[^ ^8^ ^]^ to delineate the impact of isotopic variations on organismal growth,^[^ ^9^, ^10^ ^]^ and to characterize disease mechanisms in molecular detail.^[^ ^11^, ^12^ ^]^

Protein abundance and solubility are two highly important dimensions of analysis in proteomics. With the emergence of higher multiplexing power of tandem mass tag (TMTpro‐16 and −18plex sets,^[^ ^13^, ^14^ ^]^ and the promise for higher multiplexing potential in the future, it was tempting to integrate redox proteomics in the arsenal of these chemical proteomics tools for three‐facet (3f) analysis of the proteome within the same multiplexed set. The redox state of proteins is arguably one of the most important attributes of a given proteome. Indeed, redox proteomics, although not a recent technique,^[^ ^15^, ^16^ ^]^ is rapidly gaining the attention of the proteomics community.^[^ ^17^, ^18^ ^]^

Reactive oxygen species (ROS) are metabolic or signaling molecules that exert their effects by oxidizing protein amino acid residues, particularly Cys,^[^ ^19^ ^]^ which can lead to modulation of protein activity or dysfunction.^[^ ^20^, ^21^ ^]^ Unbiased proteome‐wide mapping of redox‐regulated Cys residues, i.e., redox proteomics analysis, is therefore essential for understanding ROS‐mediated events. Advances in mass spectrometry‐based proteomics have brought about several strategies in this area, but the coverage of cysteinome remained greatly inferior compared to expression proteomics or protein solubility/stability analyses (i.e., hundreds of analyzed proteins vs >5000 proteins in a typical case). With Cys constituting 2.3% of the proteome's amino acids,^[^ ^22^ ^]^ ≈20% of all tryptic peptides contain at least one Cys; therefore, redox proteomics has significant unrealized potential in terms of proteome coverage.

One of the pitfalls of redox proteomics is that Cys residues, besides being in a free thiol or disulfide state, may undergo several reversible and irreversible modifications, such as sulfenylation, sulfinylation, sulfonylation, nitrosylation, glutathionylation, cysteinylation and persulfidation,^[^ ^23^ ^]^ which complicates peptide detection by database search. Furthermore, in most tissues and organelles, only ≈10% of reversibly modified Cys residues are estimated to be oxidized on average,^[^ ^24^ ^]^ and the low abundance often precludes the oxidized peptide from being identified, and even more so, quantified in an LC‐MS/MS experiment. Since most redox proteomics techniques are based upon simultaneous quantification of both reduced and oxidized version of a given Cys‐containing peptide to enable calculation of the oxidation occupancy,^[^ ^1^ ^]^ these techniques have achieved a rather low coverage. Extensive bioinformatics (e.g., imputing missing values), reverse labeling strategies with light and heavy isotopes, and retention time matching provide only partial remedy.^[^ ^25^ ^]^

Multiple strategies have been applied to enrich for Cys‐containing peptides using isotope‐coded affinity tags (ICAT),^[^ ^26^ ^]^ OxICAT,^[^ ^27^ ^]^ irreversible isobaric iodoacetyl tandem mass tags (iodoTMT),^[^ ^28^ ^]^ or OxiTMT,^[^ ^29^ ^]^ isotopic tandem orthogonal proteolysis‐activity‐based protein profiling (isoTOP‐ABPP),^[^ ^30^ ^]^ click chemistry,^[^ ^31^ ^]^ and Cys‐reactive phosphate tags coupled to IMAC enrichment.^[^ ^24^ ^]^ While some of these approaches provide quite decent cysteinome coverage (≥1000 proteins per analysis), the enrichment step involves labor‐intensive workflows with multiple washing steps that can result in sample loss and/or induction of spontaneous oxidation events. Importantly, upon Cys‐peptide enrichment, protein abundance information is lost, which necessitates performing expression analysis as a separate experiment.^[^ ^32^ ^]^

In a recent technique called Stable Isotope Cys Labeling with IodoAcetamide or SICyLIA,^[^ ^32^ ^]^ some of these issues have been addressed. In this technique, proteins are extracted in the presence of either light iodoacetamide (IAA) or heavy isotope‐labeled IAA to alkylate free Cys thiols in proteins by a carbamidomethyl group. After mixing equal amounts of modified light and heavy protein extracts representing two different samples under comparison, dithiothreitol (DTT) is used to reduce the reversibly oxidized thiols, which are subsequently alkylated with N‐ethylmaleimide (NEM). The ratio of the heavy to light labeled Cys serves as a proxy for comparing the levels of reduced Cys in the two samples. This technique circumvents the need to measure the oxidized protein thiols (NEM‐labeled and irreversibly oxidized peptides are simply ignored in SICyLIA) and therefore, using peptide fractionation prior to LC‐MS/MS, one can achieve a satisfactory cysteinome coverage without any enrichment. While this approach provided by far one of the best redox proteome coverage (9479 peptides in mouse cells and 4415 in tissues, corresponding to 3563 and 2168 proteins, respectively, after keeping unique peptides quantified in 3 out of the 4 replicates), under dynamic cellular conditions a separate expression proteome analysis must be performed in parallel to SICyLIA in order to account for changes in the protein abundances. In the cited work, this was achieved by dimethylating tryptic peptides using light or heavy formaldehyde/sodium cyanoborohydride. The two data sets were then matched, and the redox proteomics data were normalized by the abundances of the corresponding proteins.

Since the redox and expression samples in SICyLIA undergo different processing and labeling procedures, combining their results into a single data set can be challenging and may entail some data loss. Moreover, unless for each tryptic peptide both light and heavy dimethylated variants are quantified, missing values inevitably emerge. Another significant limitation is that only two samples can be multiplexed in redox or abundance analysis by dimethylation. In addition, heavy IAA is three orders of magnitude more expensive than light IAA (1 g of heavy IAA costs more than 1 kg of light IAA). A similar approach has also been reported, in which the reduction step after alkylation is removed to enable the quantification of various Cys modifications. This technique led to quantification of a total of 10 924 cysteine‐containing peptides in pancreatic *β*‐cells.^[^ ^33^ ^]^

Having learned from the above techniques and attempting to reach the goal of multiplexing the whole experiment into a single TMT set, we address the mentioned problems here by introducing the REX proteomics approach that integrates the redox and expression facets. We also combine REX with PISA assay and remove redundancies in expression analysis. To test this compact PISA‐REX technique, we first applied it to HCT116 cells treated with the redox‐active drug auranofin, which we have extensively characterized previously with expression, solubility, and redox proteomics.^[^ ^1^ ^]^ Subsequently, we performed a PISA‐REX investigation of interferon *α* stimulation in human monocytes, generating a resource for the community. Finally, we investigated proteome changes in pDCs isolated from wild‐type versus *Ncf1*‐mutant mice treated with IFN‐*α*, to uncover the key molecular events associated with this mutation in lupus. In all these cases, the treatments profoundly altered the redox state of the cellular proteome. With PISA‐REX we identified the targets and the mechanisms of treatments and then validated the most important findings using orthogonal approaches.

## 2 Results

### 2.1 REX with Separate Redox and Expression TMT Channels

**Figure 1:**
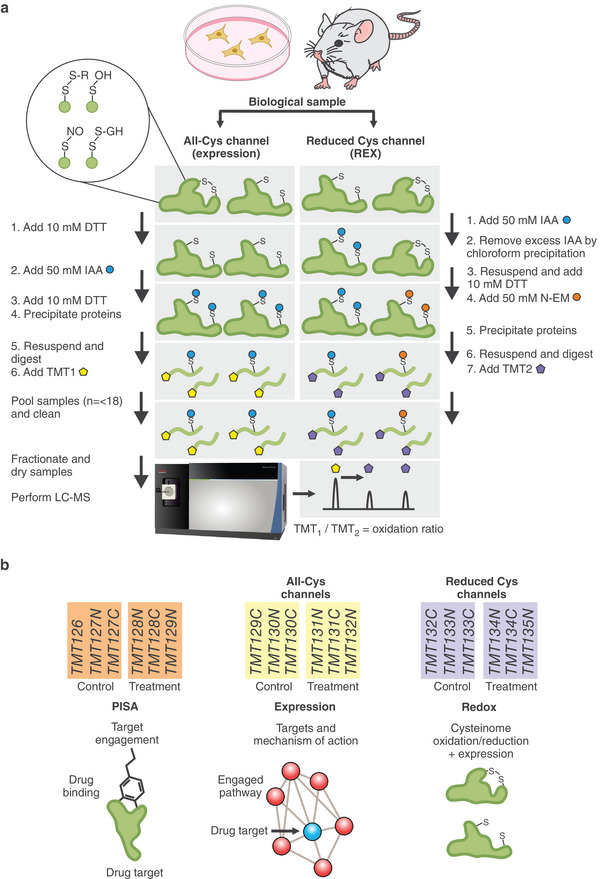
PISA‐REX workflow and experiment design. a) The REX sample preparation protocol for one condition is shown. A given biological sample is divided into two portions. One portion intended for expression analysis is processed according to the routine proteomics workflow, with full reduction of Cys with DTT and alkylation with IAA. The other portion devoted to redox analysis is first labeled with IAA, and after removal of IAA, DTT is added to reduce the disulfide bonds and reversibly oxidized Cys and block them with NEM. b) The experimental design for inclusion of PISA and REX proteomics samples within a TMTpro‐18plex set‐up. The expected information obtained from each type of analysis is shown. In biological systems where a higher variation is expected, e.g., patient samples, this experimental design can be adapted to include more replicates of any dimensions including REX.

First, we combined expression proteomics with redox proteomics using separate TMT labels (channels). For expression analysis, the whole protein lysate was reduced with DTT and then labeled with IAA, as in conventional proteomics (**Figure** **1a**). For redox analysis, the normally dominant reduced Cys thiols were labeled with conventional IAA, after which oxidized Cys residues were reduced by DTT and blocked with NEM (Figure 1a). Note that the addition of DTT at this step reduces disulfide bonds as well as sulfenylation, nitrosylation, and glutathionylation, which are also later modified with NEM. The main purpose of NEM labeling was to stabilize the newly formed thiols and prevent secondary reactions; as in SICyLIA,^[^ ^32^ ^]^ no detection of the NEM‐labeled peptides is required. As no isotope labeling is used in REX, the cost of sample preparation remains minimal. The proteins were then digested with trypsin, and tryptic peptides corresponding to control and treated samples were labeled with unique TMT labels.^[^ ^14^ ^]^ A PISA analysis was then performed as described earlier.^[^ ^4^, ^6^ ^]^ All REX and PISA samples (treated vs untreated cells in 3 replicates) are then combined within a single TMTpro‐18plex set, providing a 3‐faceted (3f) PISA‐REX experiment (Figure 1b). When performing our experiments, TMT‐pro18plex was not available, so we used TMT‐pro16‐plex (expression and REX experiments in 3 replicates and PISA in 2 replicates).

As proof of principle, we applied PISA‐REX to HCT116 cells treated with auranofin, a redox‐active compound approved for the treatment of rheumatoid arthritis. Auranofin is being repurposed in several clinical trials on ovarian cancer, lung cancer, and chronic lymphocytic leukemia (trial numbers NCT01419691, NCT01737502, NCT01747798, and NCT03456700; see www.clinicaltrials.gov). Auranofin is a specific inhibitor of thioredoxin reductase 1 (TXNRD1).^[^ ^34^ ^]^ Therefore, unlike other agents such as hydrogen peroxide, the impact of auranofin on the cell proteome is more specific, and this is why we chose auranofin as a model in this study. Furthermore, we have recently applied comprehensive chemical proteomics tools to study auranofin, including TPP, Functional Identification of Target by Expression Proteomics or FITExP (based on expression proteomics),^[^ ^35^ ^]^ and redox proteomics through sequential iodoTMT labeling.^[^ ^1^ ^]^ Therefore, to benchmark PISA‐REX (dataset 1), we replicated the same experimental conditions, i.e., for PISA and redox analysis, cells were treated with 3 µm auranofin for 2 h, while for the expression assessment, cells were treated for 48 h at IC50 concentration (1.5 µm), as measured by CellTitre Blue and MTT assays (Data S1 and S2 provide protein and peptide level data, respectively, Supporting Information).

In our previous work, the redox analysis covered only 2129 peptides belonging to 1383 proteins.^[^ ^1^ ^]^ In contrast, here (dataset 1) we quantified 98 945 peptides. Of the 18 538 Cys containing peptides, 17 089 carbamidomethylated molecules (13 907 unique sequences) were detected belonging to 5029 proteins (92.2% IAA labeling efficiency).

**Figure 2:**
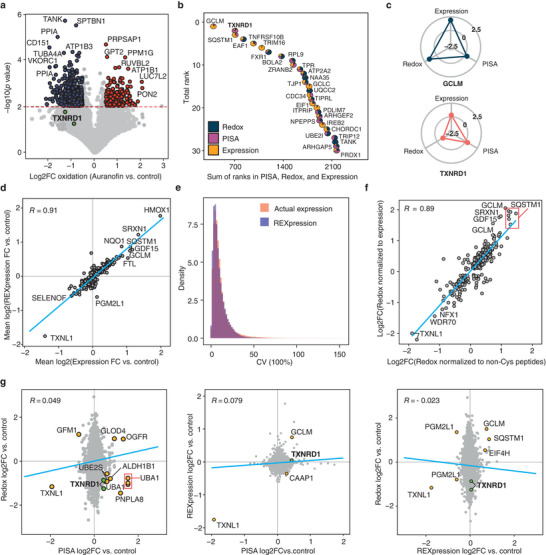
Benchmarking PISA‐REX. a) A 2 h treatment with auranofin induces massive changes in the redox state of the proteome. *P* values were calculated using a two‐sided Student's *t*‐test. Peptides with an absolute log2FC > 0.25 and *p‐*value less than 0.01 are highlighted. b) Waterfall plot of the top targets ranked across all three dimensions upon auranofin treatment (all proteins have a Fisher *p*‐value < 0.05 across all three dimensions). The pie piece size is proportional to the ranking of each protein in the respective dimension (the lower the ranking, the bigger the piece). c) Radarplots depict the redox, solubility, and expression changes of top proteins GCLM and TXNRD1 upon auranofin treatment (the radarplots range from log2FC −2.5 to 2.5; the coloring is stochastic). d) Correlation of protein abundance changes calculated using actual expression channels or those inferred from the non‐Cys peptides in REX (REXpression) in dataset 2. Only peptides passing significance in REXpression were used. Proteins with *p*‐value <0.05 across both analyses were used. e) The distribution of CV between replicates for REXpression versus expression in dataset 2. f) Correlation between FCs obtained from normalization of REX data to the expression channels versus to the sum of non‐Cys peptides from the same REX channels in dataset 2. Peptides with *p*‐value < 0.05 across both analyses were used. g) The scatterplot of different facets highlights proteins changing in different dimensions (the cognate target TXNRD1 is shown in green circles). Proteins or peptides with an absolute log2FC of > 0.25 and *p*‐value < 0.05 across both dimensions are highlighted. Expression and REX experiments were performed in 3 replicates and PISA experiments in 2 replicates.

Data processing was performed as follows. After removing the very few peptides with missing values in TMT channels, peptide abundances were calculated by normalizing the intensities of the corresponding reporter ions by the total intensity of that TMT channel. For the PISA and expression dimensions, protein abundances were calculated as the summed abundances of all peptides belonging to a certain protein (as given by Proteome Discoverer), and the fold changes (FCs) in auranofin treatment versus control were calculated. Redox analysis was performed for carbamidomethylated peptides, the abundances of which were normalized first by the total intensity of that TMT channel and then by the summed abundance of non‐Cys containing peptides from the same protein in the REX channels to adjust for the eventual protein abundance changes during treatment. Finally, the log2 values of the FCs of thus obtained values in control versus auranofin treatment were calculated (**Figure** **2a**). Note that in REX, the oxidation ratio in treated versus control samples is calculated by dividing the intensities of controls by those of the treated sample. The magnitude of changes across the expression and solubility dimensions upon auranofin treatment are also shown in Figure S1 (Supporting Information). The majority of the outliers recapitulate those identified in our previous study,^[^ ^1^ ^]^ showing the reproducibility of the techniques and robustness of the data analysis.

In each dimension of analysis, the *p*‐value was estimated using Student's *t*‐test with unequal variance based on the normalized intensities of treated versus control samples. For control of the FDR, a permutation analysis of the protein abundances between sample and control replicates was made. In total, ten rounds of permutation were performed for each dimension and thus estimated average FDR was generally below 4% in all dimensions. This analysis was also performed for all the other datasets within the study.

In PISA‐REX, a systematic and unbiased ranking system is needed to identify the most important proteins that are involved in response to a perturbation across all dimensions of analysis. We have previously introduced a robust ranking system for the deconvolution of drug targets across multiple experiment types.^[^ ^1^ ^]^ In this approach, in each individual dimension, the proteins or peptides are ranked based on their FC and, separately, based on *p*‐values (detailed in Methods). Then these ranks for all dimensions are summed together, and the proteins are sorted by the final ranking in ascending order. We believe this to be the most unbiased approach for target selection, especially across different dimensions. Applying to dataset 1 the same ranking system and coefficients of variation (CV) cutoff of 30% for protein data in all dimensions, auranofin target thioredoxin reductase 1 (TXNRD1) ranked second out of the 3252 proteins common for all dimensions, after only glutamate–cysteine ligase regulatory subunit (GCLM) (“waterfall plot” on Figure 2b; full rankings in Data S3, Supporting Information). In our previous study on auranofin, TXNRD1 ranked third among the dramatically lower number of proteins common for all three dimensions (232 proteins) due to the low depth of conventional redox proteomics analysis. Nuclear factor NF‐kappa‐B p100 subunit (NFKB2) and cysteine and histidine‐rich domain‐containing protein 1 (CHORDC1) previously found on the first and second positions received in our current PISA‐REX analysis the rankings 636 and 25, respectively.^[^ ^1^ ^]^ This discrepancy could be due to the much higher proteome coverage in the current study. While all three proteins harbored peptides with significantly changed oxidation levels, TXNRD1 was the only protein with significant changes in both solubility and expression dimensions, CHORDC1 only showed a significantly altered expression level, and NFKB2 did not show a significant change in either solubility or expression dimensions. The pie piece sizes in Figure 2b show the contribution of each dimension to the ranking of a protein of interest. For example, for TXNRD1, the most decisive dimension propelling the protein to the top of the list was solubility provided by PISA analysis (by rank), while the highest change was observed at the redox level. The log2FCs for TXNRD1 were 0.43 ± 0.017, 0.21 ± 0.031, and −1.28 ± 0.28 in the PISA, expression and REX dimensions, respectively. Radar plots in Figure 2c depict the changes of the top protein GCLM and TXNRD1 across the three dimensions.

The inhibition of TXNRD1 is known to induce the NFE2L2 (nuclear factor erythroid 2‐related factor 2) or NRF2 pathway,^[^ ^36^ ^]^ which then activates oxidative stress response genes such as GCLM and glutamate–cysteine ligase catalytic subunit (GCLC), that are involved in glutathione (GSH) synthesis.^[^ ^37^ ^]^ In agreement with that and our previous results,^[^ ^1^, ^38^ ^]^ the top 30 proteins mapped to NRF2 pathway (TXNRD1, GCLM, GCLC, PRDX1, and SQSTM1), glutathione synthase complex (GCLM and GCLC) and selenocysteine pathway (TXNRD1, GCLM, GCLC, and PRDX1). The changes across all the dimensions for these proteins are summarized in Data S3 (Supporting Information). These results provide proof of principle for the PISA‐REX approach.

To test whether protein abundances can also be deduced from the REX channels, thus allowing for a more compact experiment, we performed an additional LC‐MS/MS analysis (dataset 2), where PISA, redox, and expression dimensions were all profiled after 24 h of treatment at IC50 concentration of auranofin (Data S4 and S5 corresponding to protein and peptide level data, Supporting Information). In the dataset 2, we quantified 92 023 peptides in total. Of the 18 931 Cys containing peptides, 17 444 carbamidomethylated molecules were quantified (92.1% labeling efficiency) belonging to 3921 proteins. Expression FCs (auranofin vs control) calculated based on the abundance information derived from non‐Cys containing peptides (hereafter called REXpression) correlated well with those calculated based on the actual expression channels (R = 0.91) (Figure 2d). The CVs of the conventional protein expression and REXpression were also very similar (Figure 2e), both in terms of the median value (8% and 7%, respectively) as well as mean values (12% and 10%, respectively). Note that both parameters here are in favor of REXpression, which was also noted for the other datasets (see below).

In combined REXpression ranking (24 h, dataset 2) with PISA and redox rankings (2 h, dataset 1), TXNRD1 retained one of the top positions among 2814 proteins (full rankings in Data S6, Supporting Information). Furthermore, a good correlation (R = 0.89) was observed between the redox data normalized by REXpression versus those normalized by actual expression channels (Figure 2f). Therefore, we concluded that the PISA‐REX experiment can be further compacted by excluding the expression channels and using REXpression instead. Since such an approach leads in general to lower CVs, throughout this paper we will use REXpression data for protein abundances wherever applicable, even when the conventional expression is available in “all‐Cys channels” as well.

While systematic ranking is the best and most unbiased approach for identification of the top targets, scatterplots of different dimensions can also be used for visualizing outliers. We then investigated the correlations between the three dimensions of analysis. Figure 2g shows only weak correlations among them in our second PISA‐REX dataset, proving the orthogonality of these dimensions for the proteins not involved in the drug action mechanism. However, the outliers in these plots are not random proteins. As we have shown before, protein solubility/stability changes can be linked to oxidation and reduction of Cys residues.^[^ ^1^ ^]^ Thus, the outliers in the PISA versus REX scatter plot could be rich with mechanistic proteins. Consistent with that, the cognate target of auranofin TXNRD1 was among the significantly shifting proteins in two dimensions. Furthermore, thioredoxin‐like protein 1 (TXNL1) was the most significant outlier in all three dimensions. Our previous studies have shown extreme downregulation of TXNL1 upon auranofin treatment^[^ ^38^ ^]^ and concluded that this protein might be a substrate of TXNRD1.^[^ ^7^ ^]^ Here we found that the redox state of TXNL1 is also modulated in response to TXNRD1 inhibitor auranofin. Interestingly, a very recent study showed that TXNL1 is a redox‐active thioredoxin‐like protein.^[^ ^39^ ^]^

Consistent with the earlier observations that detection of the less abundant oxidized Cys‐peptides is more challenging, the number of detected peptides modified with NEM was substantially lower than the number of IAA‐modified peptides (1848 vs 17 089 in dataset 1 and 2458 vs 17 444 in dataset 2). The oxidation ratios based on the NEM‐modified peptides did not anti‐correlate well with those calculated based on carbamidomethylated peptides, and this result was consistent for all the analyzed datasets in the paper. The lack of the statistical link could be partially due to the poor accuracy in the measurement of low‐abundant NEM‐modified peptides as well as the presence of many different Cys modifications that can distort the estimation of oxidation ratios based on merely NEM‐modified peptides. Especially, reversible Cys modifications such as sulfenylation, nitrosylation, and glutathionylation will be reduced with DTT in our protocol and subsequently labeled with NEM. Therefore, the lack of an anti‐correlation is not surprising.

### 2.2 Interferons Remodel the Monocyte Proteome in all Three Dimensions

**Figure 3:**
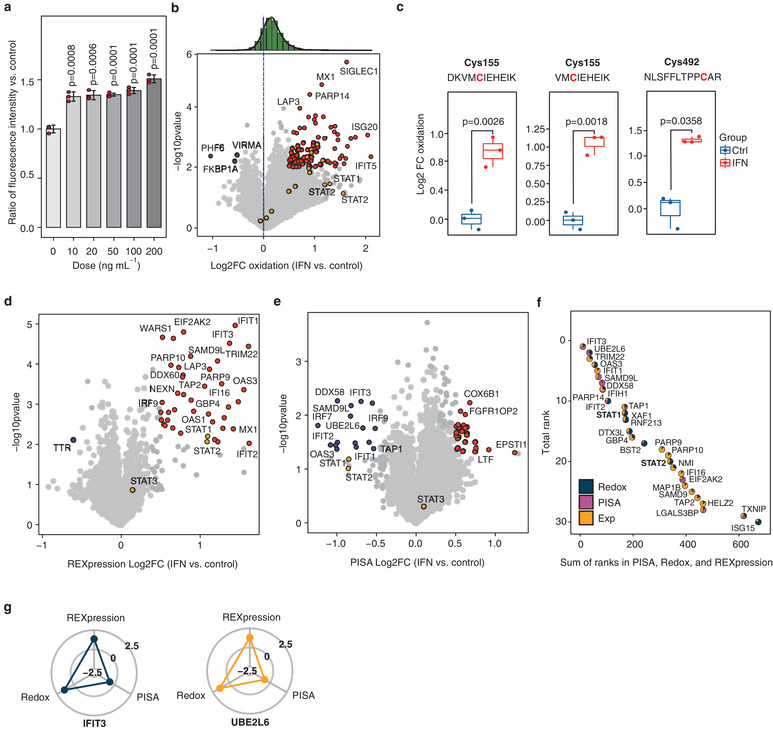
IFN‐*α* induces massive oxidation and upregulation of cellular proteins, along with changes in protein solubility. a) The extent of ROS generation across different IFN‐*α* concentrations (data are represented as mean ± SD). b) Redox changes in the proteome upon IFN‐*α* treatment. Peptides with an absolute log2FC > 0.5 and *p*‐value less than 0.01 are highlighted. c) The oxidation of three STAT1 peptides upon IFN‐*α* treatment. Boxplots: Center line‐median; box limits contain 50% of data; upper and lower quartiles, 75% and 25%; maximum‐greatest value excluding outliers; minimum‐least value excluding outliers; outliers‐more than 1.5 times of the upper and lower quartiles. Expression d) and solubility/solubility e) changes in the proteome upon IFN‐*α* treatment. Proteins with an absolute log2FC > 0.5 and *p*‐value less than 0.01 or 0.05 are highlighted in panels d and e. f) Top ranking proteins across all three dimensions (all proteins have a combined Fisher *p*‐value < 0.05). The pie piece size is proportional to the ranking of each protein in the respective dimension—larger sector for higher ranking. g) The change in the redox state, solubility, and expression of top‐ranking proteins. The radarplots range from log2FC −2.5 to 2.5. All experiments were performed in 3 independent biological replicates (PISA assay in 2 replicates), and *p*‐values were calculated using a two‐sided Student's *t*‐test.

Next, we applied PISA‐REX to investigate proteome remodeling by IFNs, signaling proteins that constitute the first line of defense against viral infections in mammals.^[^ ^40^ ^]^ IFNs interfere with viral replication by activating immune cells through the induction of transcription of hundreds of IFN‐stimulated genes (ISGs) leading to a massive proteome response.^[^ ^41^ ^]^ A recent study has applied expression proteomics and protein correlation profiling in combination with size exclusion chromatography to investigate the dynamic rewiring of the human interactome by IFN‐*β* signaling.^[^ ^42^ ^]^ However, to date, no study has been performed on the IFN‐induced proteome rewiring in the redox and protein solubility/stability dimensions. Therefore, here we applied PISA‐REX to THP1 monocytes treated with IFN‐*α*. As IFNs are known to induce oxidative stress,^[^ ^43^ ^]^ we assessed ROS production across a series of IFN‐*α* concentrations. Upon 10 ng mL^−1^ IFN‐*α* treatment, we observed a ≈30% increase in ROS production compared to untreated controls, without any discernible effect on cell viability (**Figure** **3a**). For PISA‐REX analysis we chose this concentration, which was also used in previous studies.^[^ ^42^ ^]^ The PISA‐REX data for the protein and peptide levels on THP1 cells treated with IFN‐*α* for 16 h can be found in Data S7 and S8 (Supporting Information). Of the 20 445 quantified Cys containing peptides, 18 863 were carrying the carbamidomethyl modification (12 957 unique sequences; labeling efficiency of 92.3%), mapping to 4315 proteins.

As expected, IFN‐*α* treatment induced massive oxidation of cellular proteins, denoted by the asymmetry of the density plot in Figure 3b. While it is known that redox signaling triggers and shapes the immune response including interferon signaling,^[^ ^44^ ^]^ a comprehensive account of these signaling events at the molecular level was not available. Some of the top proteins, such as STAT1, XAF1, RNF123, and PRDX4 were already known to be redox‐related. For instance, the redox state of Cys324 and Cys492 residues in STAT1 can be regulated by S‐glutathionylation.^[^ ^45^ ^]^ Here we confirmed the oxidation of Cys492 and additionally found oxidation of Cys155 (Figure 3c), supported by observation of five peptides (with two different sequences) containing this Cys. Encouragingly, our redox proteomics approach has estimated very similar oxidation ratios of the two peptides with different sequences covering Cys155 (Figure 3c). In XAF1 which is known to be transcriptionally activated by oxidative stress,^[^ ^46^ ^]^ we found oxidation of Cys112 upon IFN stimulation.

To determine whether REX consistently estimates redox changes for a specific site on a global scale, we first filtered peptides to ensure they were unique to each protein. We then retained only those peptides that contained a single Cys site and selected proteins that had at least two peptides covering the same Cys residue. Proteins with an average *p‐*value (across all relevant peptides) lower than 0.05 were retained, and we calculated the average FCs and standard deviations (SDs). 25 proteins met these criteria, with peptides covering specific Cys residues showing consistency and an SD below 0.3 in all cases. For the top 12 proteins, the positions of the specific Cys sites and the oxidation ratios for peptides covering the same sites are shown in Figure S2 (Supporting Information).

As expected, the IFN‐*α* treatment also led to changes in the REXpression and solubility of many known and putative ISGs (Figure 3d,e; the scatterplots of the three dimensions are shown in Figure S3a, Supporting Information). The top 30 proteins ranked across the three dimensions are shown in Figure 3f (full rankings in Data S9, Supporting Information). The top protein out of 3757 proteins shared among the three dimensions was IFIT3, an interferon‐induced protein with tetratricopeptide repeats 3. The fifth top protein was IFIT1, an interferon‐induced protein with tetratricopeptide repeats, while the eighth was IFIH1, an interferon‐induced protein with helicase C domain 1. STAT1 and STAT2, transcription factors of IFN response,^[^ ^47^, ^48^ ^]^ ranked 12th and 20th, respectively. At least 14 of the top 30 proteins mapped to IFN signaling (*p* = 4.62e−17). For these two top proteins, redox and REXpression were the two defining dimensions (Figure 3g).

Similar to the auranofin dataset, REXpression FCs here correlated well (R = 0.94) with the FCs obtained from the actual expression channels (Figure S3b, Supporting Information). The CVs between the replicates for REXpression were again lower than for expression (10.3% vs 12.3% for medians, and 12.8% vs 15.8% for average values), confirming that REXpression gives a more precise estimate for protein abundance changes.

### 2.3 pDCs with *Ncf1*‐Mutation Respond Differently to IFN‐*α*

Next, we applied PISA‐REX to study the role of NCF1 in a murine model of systemic lupus erythematosus (SLE). SLE is a chronic autoimmune disease predominantly observed in women of childbearing age, affecting 0.1% of the global population.^[^ ^49^ ^]^ SLE patients manifest overactivated type I IFN signature and high expression of ISGs, which are reflective of disease activity and severity.^[^ ^49^ ^]^ ROS is considered to have a regulatory role in the development of SLE, similar to other inflammatory and autoimmune diseases.^[^ ^50^ ^]^

Neutrophil cytosol factor 1 (NCF1) is a subunit of the NADPH oxidase 2 (NOX2) complex, which plays a crucial role in ROS production to protect against invading pathogens. The dysfunctional *NCF1*‐339 allele, which impairs the function of the NOX2 complex, is the major genetic association for SLE, with regards to odds ratio and allelic frequency.^[^ ^51^, ^52^ ^]^ Dysfunctional NCF1 affects ROS production and is strongly associated with SLE in humans and mouse models.^[^ ^12^ ^]^ Our previous study has found pDCs as the key immune cell type driving SLE pathogenesis.^[^ ^12^ ^]^ ROS deficiency enhances the generation, accumulation, and function of pDCs, which exacerbates pristane‐induced and spontaneous lupus. IFN‐*α* is known to autocrinally control pDC functions in cellular development, differentiation, maturation, and survival.^[^ ^53^, ^54^ ^]^

To uncover the molecular phenomena by which ROS affects pDC function in NCF1‐dependent development of lupus, we isolated pDCs from the bone marrow‐derived cells of wild type (wt) versus *Ncf1*‐mutant (*Ncf1^m1j/m1j^*) B10.Q mice. Subsequently, the cells were stimulated with 500U mL^−1^ IFN‐*α* for 20 h in culture. Since the cells were derived using magnetic bead sorting with an anti‐B220 antibody, the sorted population comprised mature pDCs and CCR9^−^pDC precursors, both exhibiting strong IFN‐*α* responses.^[^ ^55^ ^]^ Due to the point mutation in the intron7 of *Ncf1* in our model, p47phox_Δ228–235 is expressed at a low level with eight amino acid residues being deleted, leading to functional impairment of NCF1 function.^[^ ^56^ ^]^

**Figure 4:**
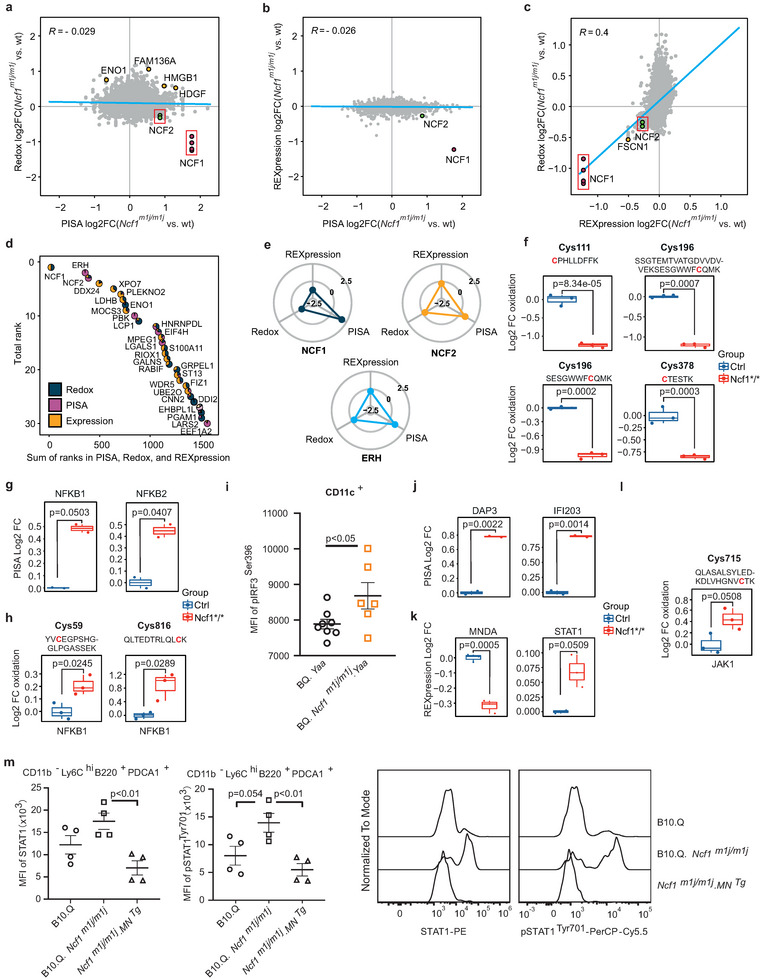
PISA‐REX for studying a mouse model of lupus. a–c) Changes in the redox state, solubility, and expression level of proteins in pDC cells isolated from *Ncf1^m1j/m1j^* mice versus wt littermates stimulated with IFN‐*α* in cell culture. The highlighted outliers have an absolute log2FC higher than 0.5 and *p*‐value less than 0.05. NCF2 is added due to its relevance to NCF1. d) The top targets ranked across three dimensions. The pie piece size is proportional to the ranking of each protein in the respective dimension. e) The changes in the redox state, solubility and expression level of top targets. The radarplots range from log2FC −2.5 to 2.5. The f) redox state of four NCF1 peptides, g) the change in the solubility of NFKB1 and NFKB2, and redox state of NFKB1 h) in *Ncf1*‐mutant versus wt cells stimulated with IFN. i) Phosphorylation of IRF3 in dendritic cells within peripheral blood from 3‐month‐old BQ.*Yaa* (*n* = 8) and BQ.*Ncf1^m1j/m1j^.Yaa* (*n* = 6) mice. j) The differential solubility, k) REXpression, and l) redox state of proteins as representative ISGs, in *Ncf1*‐mutant versus wt cells stimulated with IFN. m) Expression of STAT1 and p‐STAT1 in pDCs within peritoneal exudate cells from B10.Q, B10.Q.*Ncf1^m1j/m1j^*, and *Ncf1^m1j/m1j^.MN^tg^* mice (of each *n* = 4) at day 3 post pristane injection. Representative histograms are presented. Results are shown as mean ± SEM. Statistical significance is determined by the Two‐tailed Mann–Whitney U test in (i) and one‐way analysis of variance with Tukey's multiple comparison test in (m). For all other panels, significance is calculated by two‐sided Student's *t*‐test with unequal variance. All experiments were performed in at least 3 independent biological replicates (PISA assay in 2 replicates), unless otherwise specified. Boxplots: Center line‐median; box limits contain 50% of data; upper and lower quartiles, 75% and 25%; maximum‐greatest value excluding outliers; minimum‐least value excluding outliers; outliers‐more than 1.5 times of the upper and lower quartiles.

The PISA‐REX data corresponding to protein and peptide levels can be found in Data S10 and S11 (Supporting Information). In total, 9670 carbamidomethylated peptides were quantified without missing values. The volcano pots and scatterplots of different dimensions are shown in Figure S4 (Supporting Information) and **Figure** **4**a–c, respectively. The overall oxidation rate was higher in the *Ncf1*‐mutant cells versus wt, indicating that the mutation affects the response to IFN‐*α* treatment on the redox level (Figure 4a). The median CVs for REXpression and expression were 8.1% and 8.6%, respectively (corresponding averages being 10.3% and 10.5%). The waterfall plot of the top‐ranking proteins is shown in Figure 4d (full rankings in Data S12, Supporting Information). In the absence of eight residues in the NCF1 SH3_B_ domain, which impairs NCF1 translocation to the membrane, significant changes in the solubility, expression, and the redox state of this protein were induced under IFN‐*α* treatment conditions (Figure 4e). Interestingly, NCF2, another member of the NOX2 complex, behaved similarly. Three different NCF1 Cys residues were reduced in *Ncf1*‐mutant pDC cells upon IFN stimulation compared to wt cells (Figure 4f). Encouragingly, we estimated very similar oxidation ratios of the two peptides with different sequences covering Cys196 (Figure 4f).

Earlier studies have shown that pDCs with the *Ncf1* mutation produce higher levels of IFN‐*α*, partially through the STING pathway.^[^ ^12^ ^]^ Upon stimulation by cytosolic DNA, STING recruits NF‐kB and IRF3 to undergo phosphorylation and activation by TBK1, leading to the production of type I IFNs and other cytokines.^[^ ^57^ ^]^ PISA‐REX data analysis showed that upon IFN stimulation, the solubility of NFKB2 and NFKB1 was higher in *Ncf1*‐mutant cells versus wt (Figure 4g) and Cys59 and Cys816 from NFKB1 were more oxidized (Figure 4h). While the loss of IRF3 solubility in PISA was not statistically significant, we observed increased phosphorylation of IRF3 in dendritic cells from peripheral blood of *Ncf1*‐mutant mice in the spontaneous lupus model (Figure 4i).

The *Ncf1* mutation was also found to regulate the downstream signals mediated by IFN‐*α*. For example, *Ncf1* mutation status affected the solubility of DAP3 and IFI203 (Figure 4j), expression of MNDA and STAT1 (Figure 4k), and redox state of NFKB1 (Figure 4h) as well as JAK1 (Figure 4l). Our data confirm the regulation of STAT1, a transcriptional factor for ISGs, at the early stage of pristane‐induced lupus. pDCs from *Ncf1*‐mutant mice exhibit higher levels of STAT1 expression and phosphorylation compared to wt mice, while the levels in *Ncf1^m1j/m1j^.MN^Tg^* mice, where Ncf1 is specifically restored in pDCs and monocytes/macrophages, are comparable to wt (Figure 4m).

These results are consistent with our previous finding that NCF1 deficiency enhances the JAK1/STAT1 pathway, resulting in higher expression of ISGs and promoting lupus in both mice and humans.^[^ ^12^, ^51^ ^]^ A curious observation is the behavior of the “enigmatic” protein Enhancer of Rudimentary Homolog or ERH^[^ ^58^ ^]^ that largely mimics that of NCF1 and NCF2 (Figure 4e). Erh is involved in protein complexes related to pyrimidine metabolism, acts as a transcriptional repressor, and participates in cell cycle regulation.^[^ ^59^ ^]^ The involvement of this protein in autoimmune diseases warrants further studies.

### 2.4 REX can Uncover Irreversible Cysteine Modifications and Methionine Oxidation

The application of DTT in our protocol reduces modifications such as sulfenylation, nitrosylation, and glutathionylation. However, REX could still be used to analyze a number of irreversible Cys modifications. For example, sulfonic acid variants of Cys residues will not be reduced by DTT, and sulfinic acid variants can be partially reduced. To test if REX can be used to study such modifications, we subjected one dataset (auranofin dataset #2) to an additional search where sulfinylation (dioxidation), sulfonylation (trioxidation), and addition of NEM were included as variable Cys modifications. The search uncovered 298 peptides with sulfinylation, 411 with sulfonylation, and 2496 with NEM modifications with no missing values in the REX channels (Data S13; the corresponding plots for some of the modifications are shown in Figure S5, Supporting Information).

Methionine (Met) residues can also be oxidized. After normalizing by the total peptide intensities in each channel, the intensity of oxidized Met‐containing peptides was normalized to the summed intensity of all the non‐Cys and non‐Met‐containing peptides from the same proteins in the REX channels. Then the oxidation ratio was calculated by dividing the normalized intensity in the treatment versus the control group and the results are visualized in Figure S6, Supporting Information) and summarized in Data S14 (Supporting Information). In total, we could quantify ≈2000–4000 oxidized Met‐containing peptides in different datasets.

## 3 Discussion

Here, we developed a novel redox proteomics strategy that provides 6–9 times higher multiplexing (compared to iodoTMT labeling and SICyLIA, respectively), ≈25× lower cost per sample (compared to iodoTMT labeling or labeling with heavy IAA), and unprecedented coverage of the cysteinome. The throughput might further increase with TMTpro‐32plex becoming commercially available. Importantly, redox analysis using REX had an average overall CV below 14%, which is comparable to other types of proteomics analyses.^[^ ^60^, ^61^ ^]^ It should also be considered that variation in the measurement of peptides is generally higher than proteins, where abundance information is obtained from multiple peptides. Furthermore, we showed that the non‐Cys‐containing peptides in the REX channels can be used to extract protein abundance information (REXpression) that highly correlates with routine expression analysis. This makes PISA‐REX more straightforward, circumventing the need to perform additional expression proteomics experiments under dynamic conditions, that can lead to data loss. Furthermore, FCs calculated based on REXpression gave consistently lower CVs than those calculated from expression, which might be explained by the complex modification landscape of Cys residues that could potentially skew protein abundances in the expression channels. Therefore, unlike most redox proteomics strategies, REX can yield redox and abundance information simultaneously. REXpression will be particularly useful in future applications such as redox proteomics on small sample amounts and can potentially be used in single cell proteomics.^[^ ^62^, ^63^ ^]^

Since REX can provide simultaneous redox and expression level information, only 12 TMT channels can accommodate the PISA‐REX analysis in 3 replicates. For even more robust statistics, PISA and REX can each be performed in 4 replicates for 2 conditions using TMTpro‐16plex. For biological samples where a higher level of heterogeneity might exist between different individuals, higher number of replicates are advised (although 3 replicates were enough to study the proteome of cells isolated from our mouse model). As REX provides protein abundance information as well, up to 9 replicates can be accommodated within a given TMTpro‐18plex set without losing abundance information. Under such conditions, PISA experiments can be performed separately and integrated into PISA‐REX. TMT reagents with higher multiplexing potential (e.g., 32plex) will allow for a more interactive and inclusive experimental design.

Upon benchmarking REX against our comprehensive chemical proteomics on auranofin,^[^ ^1^ ^]^ we integrated this tool with PISA assay, introducing a possible industry standard proteomics tool. We applied such a PISA‐REX tool to several model systems, showcasing target deconvolution for drugs and biological agents such as IFNs and disease mechanisms in a murine model of lupus. We showed 3D proteome remodeling upon IFN‐*α* treatment, revealing ISGs that are redox modulated and those that undergo solubility transitions on top of expression changes. By applying REX to a murine model of lupus, we demonstrated that upon IFN‐*α* stimulation, mutant *Ncf1*, an important gene with polymorphisms in the etiology of this disease, is strongly modulated at the redox, solubility, and expression levels compared to the wt mice. Furthermore, we showed the involvement of STAT1 and NFKB signaling in the molecular events in pathways dependent on NCF1. ROS deficiency enhances the generation, accumulation, and function of pDCs, which are the key immune cell types driving SLE pathogenesis. Here, we uncovered the molecular pathways by which IFN‐*α* controls pDC functions.

In this paper, we mostly focused on the quantification of the dominant reduced form of Cys. Furthermore, we show that other types of non‐reducible Cys modifications can be captured in REX. As the forms of Cys oxidation upon different treatments can vary, enrichment strategies might be needed for analysis of each specific type of Cys oxidation, as previously described for nitrosylation.^[^ ^64^ ^]^ In this paper, we chose DTT and IAA, as the most extensively used strong reducing and alkylating agents, respectively. IAA has a high labeling efficiency as shown in several instances in the current study. Furthermore, we used the IAA and NEM pair, so that our results could be compared with the benchmark technique SICyLIA.^[^ ^32^ ^]^ However, several studies have shown that the choice of alkylating agents can potentially impact the identification and quantification of specific forms of Cys oxidation.^[^ ^65^, ^66^, ^67^ ^]^ This is because no electrophile reacts fast with all the available free Cys residues. Therefore, future studies should investigate the impact of using different alkylating agents or perhaps even combine such agents for more comprehensive redox proteomics analysis.

REX can quantify the oxidation/modification level of ≈20 000 Cys containing peptides in a given cell line. When profiling three treated cell lines, we quantified ≈35 000 Cys containing peptides using REX (unpublished data). Even large‐scale studies performed using multiple TMT sets can uncover only ≈45 000 Cys‐containing peptides.^[^ ^38^ ^]^ This is while ≈214 000 Cys residues are estimated to exist in the human proteome.^[^ ^68^ ^]^ There might be several reasons behind the quantification of only a subset of potential Cys containing peptides in individual proteomics experiments. For example, not all proteins are expressed in every cell line and tissue. In our experience, the addition of more cell lines representing other tissues drastically improves the cysteinome coverage. Furthermore, due to the stochasticity of peptide sampling in LC‐MS, the level of expression of proteins will affect the number and identity of the quantified peptides. Also, some proteins are simply not amenable to digestion with trypsin, or produce too small or too large peptides,^[^ ^69^ ^]^ limiting the overall sequence coverage in proteomics experiments. Combining different proteases might therefore provide an opportunity to enhance the cysteinome coverage by REX.^[^ ^70^, ^71^ ^]^ As mentioned above, there is also a certain level of bias with Cys accessibility and the choice of alkylating agents. It should be noted that the inclusion of PISA in the TMT sets can be considered a limiting factor in achieving the highest proteome and cysteinome coverage, as the mild cell lysis in PISA is not as efficient as in expression and REX. Altogether, we believe that REX can quantify the majority of accessible Cys containing peptides from reasonably expressed proteins in a given cell or tissue without a special enrichment process. Our unbiased ranking system coupled to pathway enrichment analysis can be used as a powerful tool to derive meaningful information from PISA‐REX.

Studying Cys modifications is of utmost importance, as such PTMs are involved in modulating protein function.^[^ ^21^ ^]^ For instance, Cys modifications and redox signaling broadly regulate the immune response,^[^ ^72^ ^]^ pancreatic β‐cells physiology,^[^ ^73^ ^]^ and an array of cellular and organismal processes. On the other hand, dysregulation of redox signaling pathways can be involved in the pathobiology of disease. For example, Parkin is an E3 ubiquitin ligase the dysfunction of which causes accumulation of protein aggregates in dopaminergic neurons.^[^ ^74^ ^]^ Several studies have shown the inactivation of Parkin through nitrosylation or sulfonylation and its potential involvement in Parkinson's disease.^[^ ^75^, ^76^, ^77^ ^]^ These examples underscore the importance of redox proteomics in studying biology and disease. We envision the widespread use of PISA‐REX in academia and industry as a robust tool for target deconvolution as well as gaining biological insight into disease mechanisms.

One limitation of REX is that fractionation is required for achieving high cysteinome coverage, as multiplexing the samples within TMT sets adds to sample complexity. The development of MS systems with higher analytical speed will reduce the time required for the analysis in the future. Specific Cys modifications such as sulfenylation, nitrosylation and glutathionylation are susceptible to reduction by DTT in our approach. When analyzing the reduced Cys samples, persulfide variants of Cys residues will also be alkylated and the addition of DTT will subsequently reduce the disulfide inside the previously alkylated persulfide.^[^ ^78^, ^79^ ^]^ While REX can quantify irreversible Cys modifications, enrichment strategies or other tailored approaches are needed to comprehensively study these, and reversible Cys modifications. For example, Li et al. avoided the reduction step after initial alkylation, which allowed for quantification of various Cys modifications (including 97 sulfinylations and 291 sulfonylations) in mouse pancreatic β‐cells treated with thapsigargin.^[^ ^33^ ^]^

## 4 Conclusion

In summary, we devised a novel high‐throughput and high‐depth redox proteomics approach integrated with expression proteomics and combined it with proteome‐wide solubility analysis. This multifaceted tool PISA‐REX can inform on the abundance, solubility, and redox state of proteins at any given biosystem in a proteome‐wide manner. Furthermore, PISA‐REX is amenable to quantifying irreversible Cys modifications and other PTMs such as phosphorylation. PISA‐REX was shown to triangulate the targets of drug and biological agents such as interferons, as well as dissecting the mechanistic proteins involved in a disease model of lupus. PISA‐REX will potentially become an industry standard proteomics tool for target deconvolution, opening new windows into the biology of health and disease.

## 5 Experimental Section

### Cell Culture

Human colorectal carcinoma HCT116 (ATCC, USA) cells were grown at 37 °C in 5% CO_2_ using McCoy's 5A modified medium (Sigma–Aldrich, USA) supplemented with 10% FBS superior (Biochrom, Berlin, Germany), 2 mm l‐glutamine (Lonza, Wakersville, MD, USA) and 100 units mL^−1^ penicillin/streptomycin (Gibco, Invitrogen). Human THP1 cells (ATCC, USA) were grown under the exact same conditions in RPMI. Low‐number passages were used for the experiments and cells were checked for mycoplasma contamination.

### Viability Assay

The viability assays were performed using CellTiter‐Blue (Promega) according to the published protocol.^[^ ^38^ ^]^ Briefly, cells were seeded at a density of 4000 per well in 96‐well plates. Adherent cells were grown for one day and treated with serial concentrations of the compounds on the next day, while the suspension‐type cells were treated 2 h after seeding. These results were also reproduced using MTT assay, as detailed before.^[^ ^80^ ^]^ IC50 was determined as the concentration inducing a 50% reduction in viability.

### ROS Production Assay

The assay was performed according to our recent protocol.^[^ ^81^ ^]^ Briefly, THP1 cells were cultured at a density of 250 000 cells in six‐well plates and stimulated separately in triplicates with 0, 10, 20, 50, 100, and 200 ng mL^−1^ IFN*α* for 16 h. After the treatment, cells were washed twice with PBS and resuspended in 2 mL PBS, and treated with 2′,7′‐dichlorofluorescin diacetate (DCF‐DA) to a final concentration of 20 µm and kept at 37 °C for 30 min. DCF‐DA was then discarded, and cells were washed twice with PBS and resuspended in 1 mL PBS again. An equal volume of cell suspension from each sample was transferred to flat‐bottom black 96‐well plates and the fluorescence was recorded at an excitation of 495 nm and emission of 527 nm. Trypan blue dye exclusion assay was also used to count the number of living cells for normalization.

### PISA‐REX Experiments

Cells were treated in 8 replicates with the vehicle or compounds according to the indicated concentrations and durations in Table S1 (Supporting Information) for different experiments. For all‐Cys and reduced Cys analyses, cells were lysed with 1% SDS, 8 m urea, Tris buffer pH 8.0 plus protease inhibitor. The cell lysates were subjected to 1 min sonication using a Branson probe sonicator with 3 s on/off pulses and a 30% amplitude. Protein concentration was measured using Pierce BCA Protein Assay Kit (Thermo), and the volume corresponding to 25 µg of protein was transferred from each sample to new tubes. For all‐Cys analysis (reduced and reversibly oxidized Cys), DTT was added to a final concentration of 10 mm and samples were incubated for 45 min at room temperature (RT). Subsequently, IAA was added to a final concentration of 50 mm and samples were incubated at RT for 1 h in the dark. The reaction was quenched by adding an additional 10 mm of DTT. Proteins were precipitated by methanol/chloroform and resuspended in 20 mm EPPS buffer pH 8.5 with 8 m urea.

For reduced Cys samples, IAA was added to a final concentration of 50 mm and samples were kept in the dark for 1 h at RT. The proteins were then precipitated by methanol/chloroform and resuspended in the same volume of lysis buffer again. DTT was added to the final concentration of 10 mm and samples were incubated for 30 min at RT (DTT addition also reduces Cys with sulfenylation, nitrosylation, and glutathionylation). NEM was added at the final concentration of 50 mm, followed by 1 h incubation in the dark at RT. After a further precipitation step, the samples were resuspended in 20 mm EPPS buffer pH 8.5 with 8 m urea.

To prevent the spontaneous oxidation events post cell lysis and before IAA treatment, the REX experiments were performed in an inert atmosphere chamber filled with argon. Furthermore, buffers were prepared in distilled deionized water, degassed under low‐pressure sonication, and bubbled with argon. The solutions were freshly made and kept on ice.

For PISA in intact cells, the previously published protocol was followed.^[^ ^4^, ^6^ ^]^ Cells were collected, centrifuged, and washed twice with PBS and then resuspended in ≈300 µL PBS. The cell suspension from each replicate was aliquoted into 10 in 96‐well plates and heated in an Eppendorf gradient thermocycler (Mastercycler X50) in the temperature range of 48–59 °C for 3 min. Samples were cooled for 3 min at RT and afterward snap frozen with liquid nitrogen and kept on ice. The cells were then lysed by five times freezing in liquid nitrogen and thawing at RT. Samples from each replicate were then combined and transferred into polycarbonate thickwall tubes and centrifuged at 100 000 g and 4 °C for 20 min. The soluble protein fraction was then collected and subjected to protein concentration measurement using the BCA assay. The reduction and alkylation were performed as detailed for all‐Cys samples. After precipitation, PISA samples were also resuspended in 20 mm EPPS buffer pH 8.5 with 8 m urea.

The rest of the protocol was the same for all three types of samples and adapted from the previous protocol.^[^ ^38^ ^]^ Urea was diluted to 4 m by adding 20 mm EPPS. Lysyl endopeptidase (LysC; Wako) was added at a 1:75 w/w ratio and incubated at RT overnight. Samples were diluted with 20 mm EPPS to the final urea concentration of 1 m, and trypsin was added at a 1:75 w/w ratio, followed by incubation for 6 h at RT.

Acetonitrile (ACN) was added to a final concentration of 20% and TMT reagents were added 4× by weight (200 µg) to each sample, followed by incubation for 2 h at RT (TMT channel assignment information is in Table S2, Supporting Information). The reaction was quenched by the addition of 0.5% hydroxylamine. Samples within each replicate were combined, acidified by TFA, cleaned using Sep‐Pak cartridges (Waters), and dried using DNA 120 SpeedVac Concentrator (Thermo). The pooled samples were resuspended in 20 mm ammonium hydroxide and separated into 96 fractions on an XBrigde BEH C18 2.1 × 150 mm column (Waters; Cat#186003023), using a Dionex Ultimate 3000 2DLC system (Thermo Scientific) over a 48 min gradient from 1% to 63% B (B = 20 mm ammonium hydroxide in acetonitrile) in three steps (1–23.5% B in 42 min, 23.5–54% B in 4 min and then 54–63% B in 2 min) at 200 µL min^−1^ flow. Fractions were then concatenated into 24 samples in sequential order (e.g., A1, C1, E1, and G1 on the 96 well plates were combined).

### LC‐MS/MS

After drying, samples were dissolved in buffer A (0.1% formic acid and 2% ACN in water). The samples were loaded onto a 50 cm EASY‐Spray column (75 µm internal diameter, packed with PepMap C18, 2 µm beads, 100 Å pore size) connected to a nanoflow Dionex UltiMate 3000 UHPLC system (Thermo) and eluted in an organic solvent gradient increasing from 3–4% to 26–28% (B: 98% ACN, 0.1% FA, 2% H_2_O) at a flow rate of 300 nL min^−1^ over 95 min. The eluent was ionized by electrospray, with molecular ions entering an Orbitrap Fusion Lumos mass spectrometer (Thermo Fisher Scientific) for auranofin samples. The rest of the samples were analyzed on either Orbitrap HF, Exploris, or Lumos (all – Thermo Fisher Scientific). Method settings are tabulated in Table S3 (Supporting Information).

### Data Processing

The raw LC‐MS/MS data were analyzed by Proteome Discoverer v2.5 (Thermo Fisher Scientific) using Mascot Server v2.5.1 as a search engine with SwissProt human (20 360 entries downloaded on 28 February 2022) or mouse (21 989 entries downloaded on 2 September 2020) and common contaminant databases. No more than two missed cleavages were allowed. A 1% false discovery rate (FDR) was used as a filter at both protein and peptide levels. For all other parameters, the default settings were used. In all analyses, reporter ions of TMTpro 16‐plex were used for peptide quantification. Cys carbamidomethylation and N‐ethylmaleimide addition, methionine oxidation, asparagine and glutamine deamidation, N‐terminal acetylation, and TMTpro (+304.207 Da) were selected as dynamic modifications. Contaminants were removed. A mutation in the Ncf1 gene (m1j) leads to a truncated NCF1 protein, thereby a non‐functional NOX2 complex, as described before.^[^ ^56^ ^]^ In the *Ncf1* dataset, no peptides containing the mutation site could be quantified; therefore, for unbiased comparison of the mutant with the wt samples, the two peptides mapping were removed to the sequence containing this site and calculated the protein abundances based on the other 12 detected peptides.

### Ethics Approval

Stockholm Animal Ethics Committee, Stockholm, Sweden, approved all the animal experiments (approval number 4516‐2017).

### Animals

C57BL/6NJ mice were originally from The Jackson Laboratory (Farmington, CN, USA). B6N.Q (C57/B6N.Q/rhd) and B10.Q (C57/B10N.Q/rhd) have been fully backcrossed into B6N and B10 genomes but with the MHC region from DBA/1 (Figure S7a, Supporting Information). The derived *Ncf1*‐mutant mouse strains on different backgrounds were designated as B6N.Q.*Ncf1^m1j/m1j^* and B10.Q.*Ncf1^m1j/m1j^*, respectively. The transgenic strain B10.Q.*Ncf1^m1j/m1j^.MN^Tg^* (herein denoted as *Ncf1^m1j/m1j^.MN^Tg^*) expresses functional NCF1 restricted to macrophages/monocytes using the human CD68 promoter.^[^ ^82^ ^]^ B6.SB‐*Yaa*/J (Stock No: 000483) from The Jackson Laboratory was fully backcrossed onto B10.Q, denoted as B10.Q.*Yaa*. The *Yaa*‐carrying strain with functional NCF1 (BQ.*Yaa*) or mutant NCF1 (BQ.*Ncf1^m1j/m1j^*.*Yaa*) was obtained by crossing B6N.Q.*Ncf1^m1j/m1j^* with B10.Q.*Yaa*. Mice were housed under specific pathogen‐free (FELASAII) conditions in individually ventilated cages with wood shaving bedding in a climate‐controlled environment with a 12 h light/dark cycle. Animal experiments were performed in a controlled way balanced for age and sex; the genotype of the mice was blinded to the investigators, following the ARRIVE guidelines.

### PDC Isolation and Stimulation

BM cells were flushed from two femurs and two tibias per mouse. After lysis of red blood cells with ammonium–chloride–potassium buffer for 1 min at RT, cells were plated at 2 × 10^6^ cells mL^−1^ in RPMI complete media containing 200 ng mL^−1^ FLT3L (BioLegend, #550704) for pDC differentiation. Cells were collected on day 9 and pDCs were magnetically isolated with biotinylated anti‐B220 mAb (BD Biosciences, clone: RA3‐6B2). Sorted pDCs were cultured at a density of 5 × 10^6^ cells per well in RPMI medium and stimulated with 500 U mL^−1^ IFN‐*α* (BioLegend, #752804) for 20 h.

### Pristane‐Induced Lupus

B10.Q, B10.Q.*Ncf1^m1j/m1j^*, and *Ncf1^m1j/m1j^.MN^Tg^* mice were injected intraperitoneally with a single dose of 500 µL pristane (Sigma–Aldrich, #P2870) (Figure S7b, Supporting Information).

### Flow Cytometry

Single‐cell suspensions were prepared from peritoneal exudates and peripheral blood from mice. After a homemade anti‐mouse CD16/CD32 FcR block (clone: 2.4G2), the cells were incubated with LIVE/DEAD Fixable Near‐IR Dead Cell Stain Kit (ThermoFisher, #L10119) and labeled with the following antibodies: anti‐CD11c (BD Biosciences, clone: HL3, PE‐Cyanine7), anti‐CD11b (BioLegend, clone: M1/70, Pacific Blue), anti‐Ly6C (BioLegend, clone: HK1.4, FITC), anti‐PDCA1 (BioLegend, clone: 927, Alexa Fluor 647), anti‐B220 (BD Biosciences, clone: RA3‐6B2, PE‐Cyanine7), anti‐STAT1 (BD Biosciences, clone: 1/Stat1, PE), anti‐pSTAT1^Tyr701^ (BD Biosciences, clone: 4a, PerCP/Cy5.5), and anti‐pIRF3^Ser396^ (Bioss Antibodies, polyclonal, FITC). For intranuclear staining, cells were fixed and permeabilized with eBioscience Foxp3/Transcription Factor Fixation/Permeabilization Kit (ThermoFisher, #00‐5521‐00). Samples were acquired using ThermoFisher Attune NxT Flow Cytometer equipped with ThermoFisher Attune NxT Software version 3.2.1, and the data were analyzed using the BD FlowJo version 10.7.1.

### Statistical Analysis

Data analysis was performed using R project versions 3.6–4.0. The raw abundances were normalized to the total channel intensity. Since under longer treatment times (>1 h) protein abundances may also change, in PISA assay, the data were normalized to protein abundances when the treatment times were matched. Redox data was analyzed at the peptide level, where the abundance of all peptides quantified in the REX channels was calculated by dividing their individual intensity by the summed intensity of that channel for a given sample. For redox analysis, the obtained abundance of Cys‐containing peptides was normalized by the summed abundance of non‐Cys peptides from the same protein. The oxidation ratio was calculated as the normalized abundance of peptide in control versus treated samples (in NEM‐modified peptide analysis, the calculation of oxidation ratio was reversed). *p*‐values for the potential target proteins were calculated by *t*‐test based on normalized abundances between control and treatments in all cases. Two‐sided unpaired *t*‐test with unequal variance was applied to calculate *p*‐values, unless otherwise specified (alpha = 0.05). Fisher's formula was used for the calculation of statistical significance across the three dimensions (merged *p*‐values). In all cases, significance was defined as *p* ≤ 0.05. Data were collected in at least 3 replicates across all experiments (PISA experiments in 2 replicates) and data is presented as mean ± SD, unless otherwise specified.

For unbiased target ranking, the proteins were individually ranked in each dimension for absolute values of the log2 of their FC and the *p*‐value and then the sum of the two ranks was calculated. For expression, ranking was done on the log2FC, as the majority of targets are upregulated.^[^ ^1^, ^38^ ^]^ Then thus obtained rankings were summed across the three dimensions, and the proteins were ranked again. The proteins with the lowest summed rank were the top proteins. We have explained this approach in detail in a previous study.^[^ ^1^ ^]^ In the scatterplots, the FC cutoff was arbitrary, and all outliers passed the stated significance.

### Network Mapping

GO pathway enrichment analysis for the top proteins was performed using the StringDB tool version 12.^[^ ^83^ ^]^

## Conflict of Interest

The authors declare no conflict of interest.

## Author Contributions

A.L. and H.L. contributed equally to this work. R.A.Z. and A.A.S. conceptualized the study; A.A.S., R.A.Z., S.P.G., X.Z., H.L., and A.V. developed the methodology and experiment design; R.A.Z., A.A.S., and R.H. organized the project, performed training, gathered resources, and acquired funds; A.A.S., H.L., J.T., and M.G. performed Proteomics experiments; H.L. performed in vivo experiments; A.L., A.A.S., A.V., and H.G. performed data analysis and visualization; A.A.S. and R.A.Z. wrote the original draft; All the co‐authors wrote, reviewed, and edited the final draft.

## Supporting information

Supporting Information

Supplemental Data 1

Supplemental Data 2

Supplemental Data 3

Supplemental Data 4

Supplemental Data 5

Supplemental Data 6

Supplemental Data 7

Supplemental Data 8

Supplemental Data 9

Supplemental Data 10

Supplemental Data 11

Supplemental Data 12

Supplemental Data 13

Supplemental Data 14

## Data Availability

The LC‐MS/MS raw data files and extracted peptides and protein abundances are deposited in the jPOST repository of the ProteomeXchange Consortium under the dataset identifiers PXD035964 (animal experiment) [https://www.ebi.ac.uk/pride/archive/projects/PXD035964] and PXD042967 for all the other datasets with no restrictions. The extracted protein abundances data and relevant outputs of data analysis are provided in Supplementary Data 1‐14.
