# Supplementary material for: Multifaceted Proteome Analysis at Solubility, Redox, and Expression Dimensions for Target Identification
Source: Adv Sci (Weinh). 2024 Aug 9;11(38):2401502. doi: 10.1002/advs.202401502 (PMC11481203; doi:10.1002/advs.202401502)
Supplement: Supplementary file 1 — Supporting Information [file ADVS-11-2401502-s009.docx]

Supporting Information

**Multifaceted Proteome Analysis at Solubility, Redox, and Expression Dimensions for Target Identification**

Amir A. Saei*, Albin Lundin, Hezheng Lyu, Hassan Gharibi, Huqiao Luo, Jaakko Teppo, Xuepei Zhang, Massimiliano Gaetani, Ákos Végvári, Rikard Holmdahl, Steven P. Gygi, Roman A. Zubarev*

**Supplementary Figures**


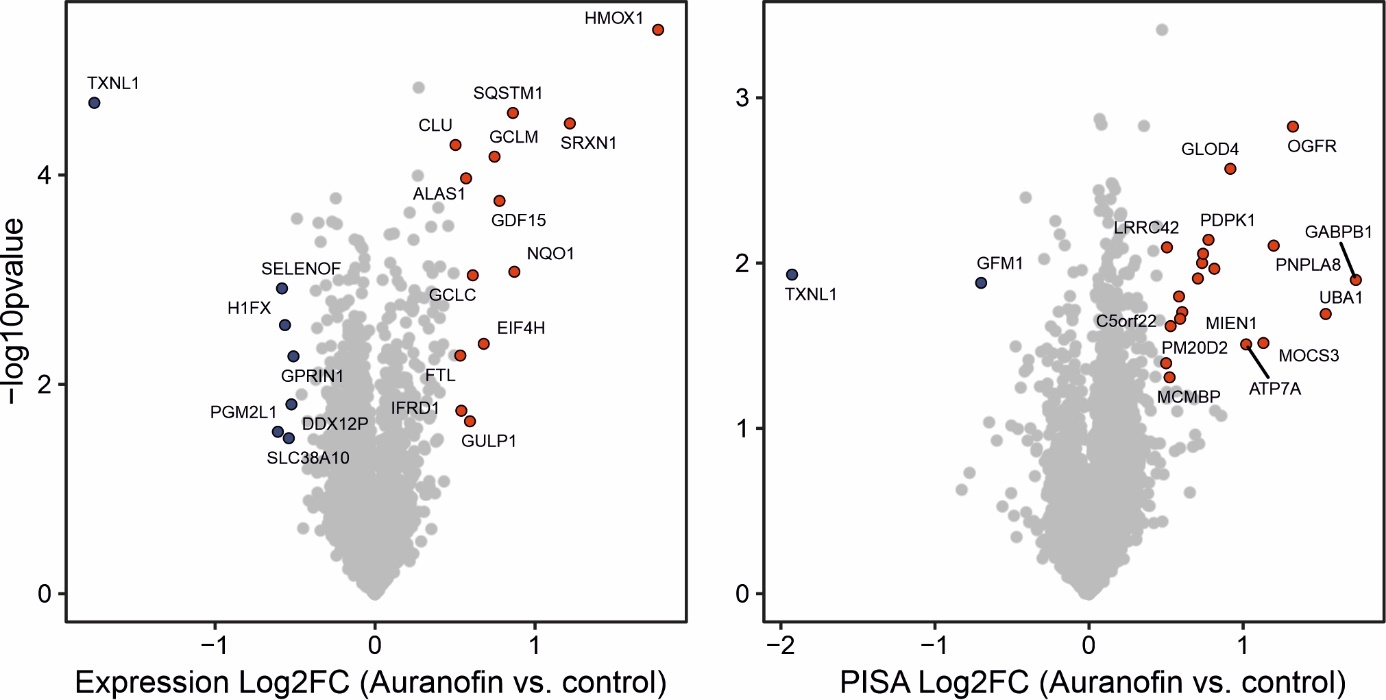


**Figure S1.** The proteome changes across expression (24h) and PISA (2h) dimensions upon auranofin treatment. The upregulated (red) and down-regulated (blue) proteins with an absolute log2FC > 0.5 and *p* value < 0.05 are highlighted. Expression and PISA analyses were performed in 3 and 2 independent biological replicates, respectively, and *p* values were calculated using two-sided Student’s t-test.


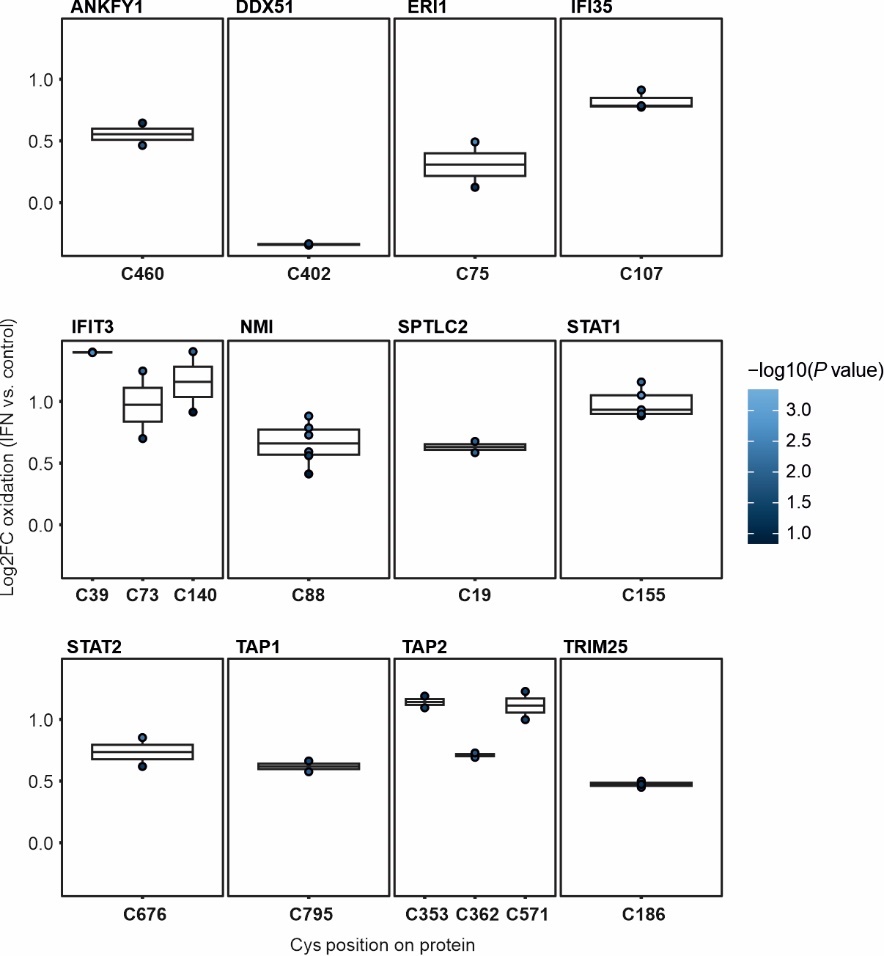


**Figure S2.** The consistency in estimating oxidation ratio of Cys-containing peptides for different peptides from the same protein covering specific Cys residues (each circle representing a peptide). The color indicates the *p* value significance level. Experiments were performed in three independent biological replicates and *p* values were calculated using two-sided Student’s t-test. Boxplots: Center line-median; box limits contain 50% of data; upper and lower quartiles, 75 and 25%; maximum-greatest value excluding outliers; minimum-least value excluding outliers; outliers-more than 1.5 times of the upper and lower quartiles.


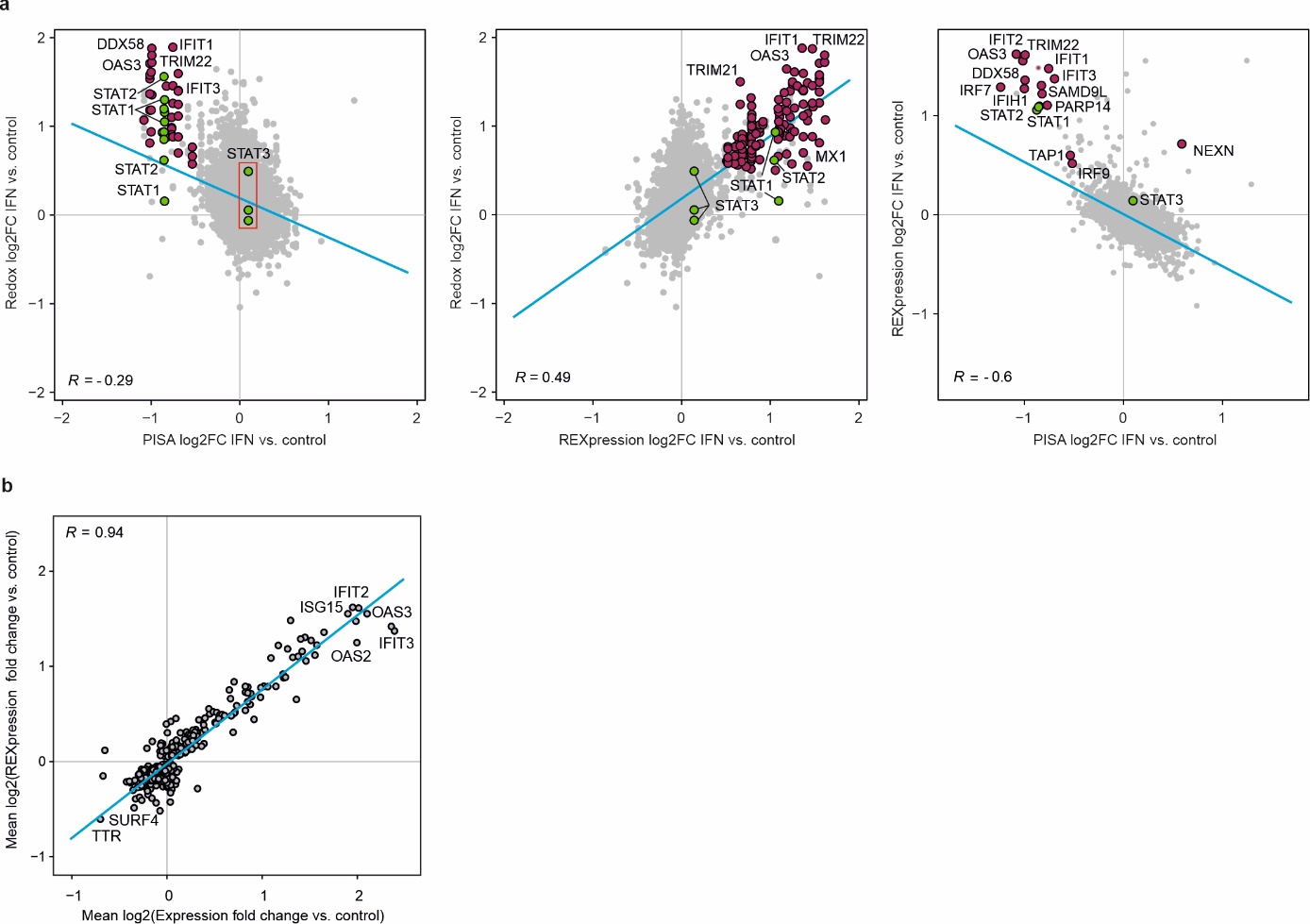


**Figure S3.** a) The scatterplots of three different dimensions highlight proteins changing across different dimensions upon IFN-α treatment. Outliers represent peptide or proteins with an absolute Log2FC of > 0.5 and a *p* value <0.05 across both shown dimensions. b) The correlation of protein FCs upon IFN-α treatment vs. control, in REXpression vs. expression. Proteins with *p* value <0.05 across both expression and REXpression were used for the analysis. Expression and REX experiments were performed in 3 and PISA assay in 2 independent biological replicates. *P* values were calculated using two-sided Student’s t-test.


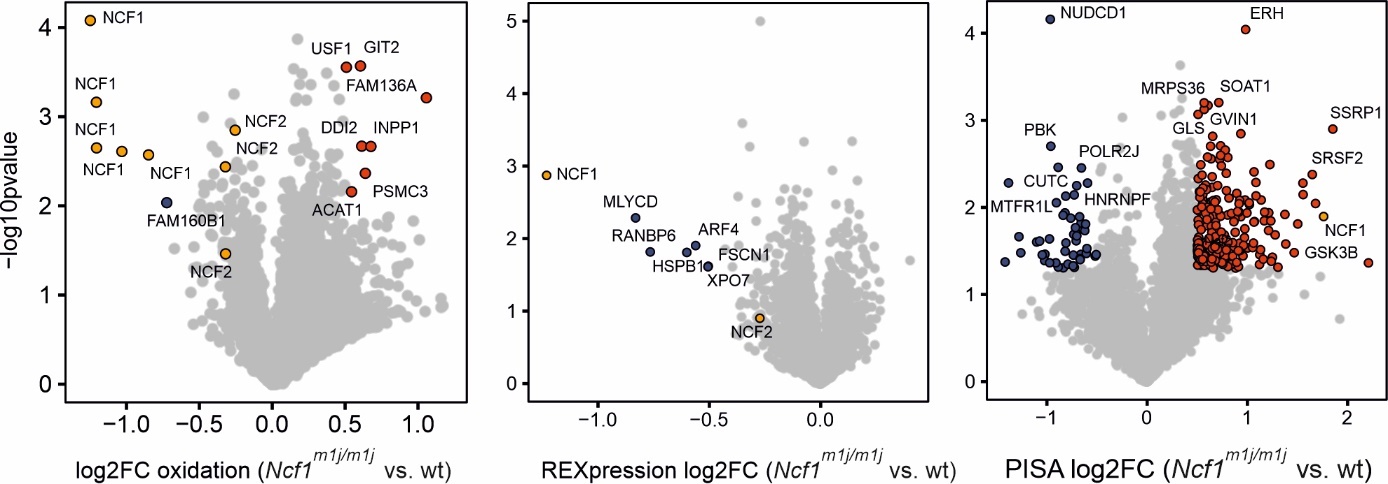


**Figure S4.** Changes in the redox state, expression and solubility level of proteins in PDC cells isolated from *Ncf1^m1j/m1j^* mice vs. wt littermates stimulated with IFN-α in cell culture. The upregulated (red) and down-regulated (blue) proteins with an absolute log2FC > 0.5 and *p* value < 0.01 are shown. NCF1 and 2 are shown with orange dots. Experiments were performed in 3 independent biological replicates (PISA assay in 2 replicates) and *p* values were calculated using two-sided Student’s t-test.


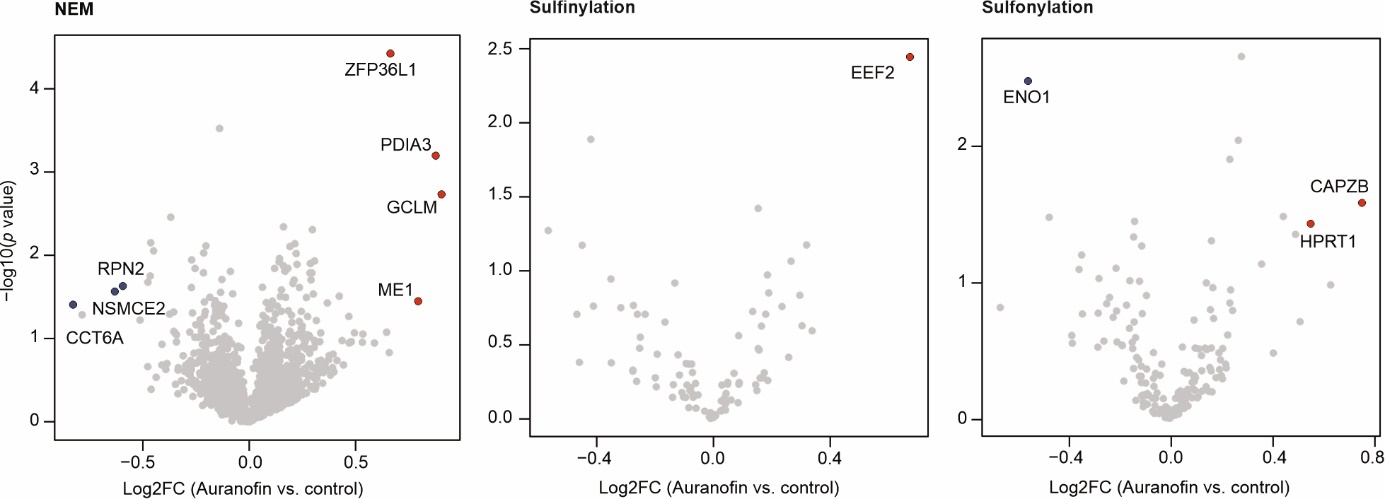


**Figure S5.** REX quantifies other types of Cys modifications upon auranofin treatment (n=3 independent biological replicates). *P* values were calculated using two-sided Student’s t-test.


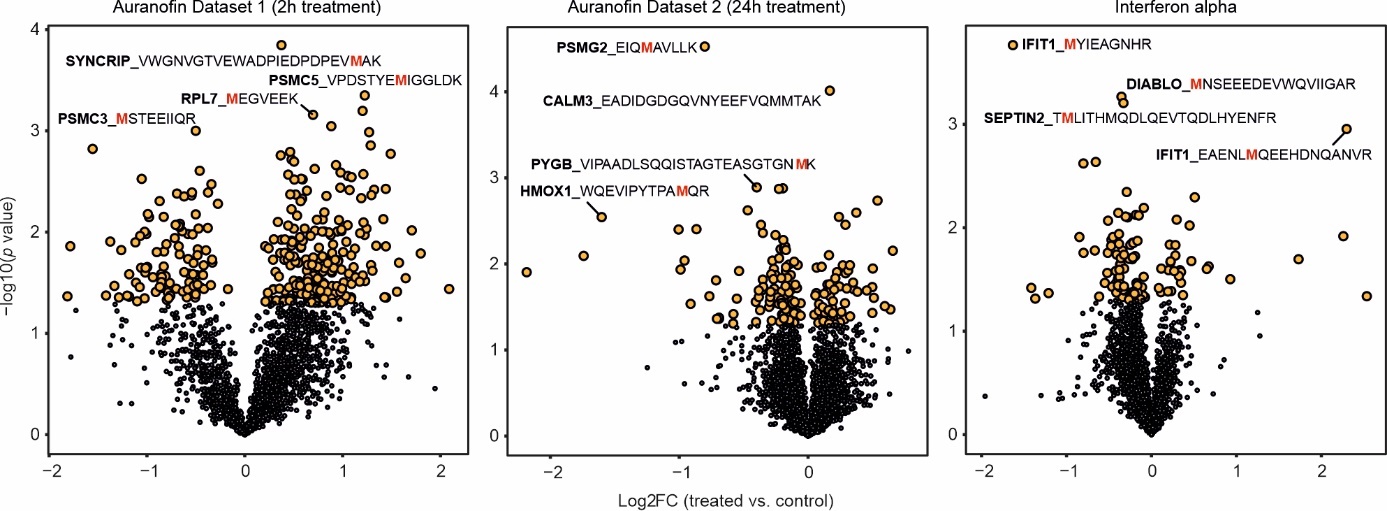


**Figure S6.** The oxidation of Met-containing peptides across the proteome under different perturbations, as calculated using the REX channels. The peptides are labeled with gene names followed by the peptide sequence (n=3 independent biological replicates).


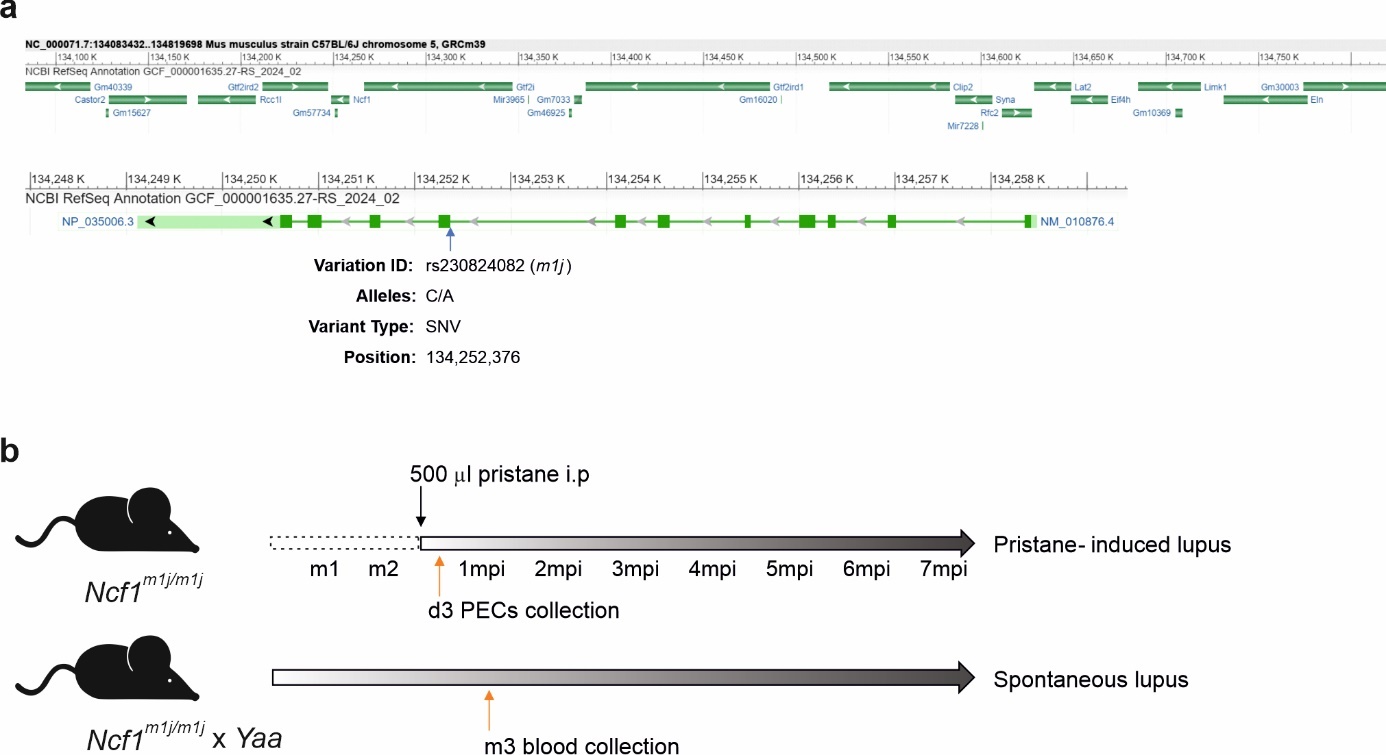


**Figure S7.** a) The animal model used in the study. b) the *in vivo* experiment set up.

**Supplementary Tables**

**Table S1.** The treatment concentrations and durations

| **Experiment** | **Cells and concentrations** | **PISA treat time** | **Expression treat time** | **REX treat time** |
| --- | --- | --- | --- | --- |
| Auranofin #1 | PISA and REX at 3 μM and expression at 1.5 μM | 2 h | 48 h | 2 h |
| Auranofin #2 | HCT116 (1.5 μM) | 24 h | 24 h | 24 h |
| Interferons α | THP1 (10 ng/mL) | 16 h | 16 h | 16 h |
| Interferons α | PDC cells from mutant and wt mice (500U/ml) | 20 h | 20 h | 20 h |

**Table S2. Experimental design for the PISA-REX experiments.** The rows correspond to the TMT labels 1-16

| **TMT channel** | **Sample** |
| --- | --- |
| 1 | PISA control |
| 2 | PISA control |
| 3 | PISA treatment |
| 4 | PISA treatment |
| 5 | Expression control |
| 6 | Expression control |
| 7 | Expression control |
| 8 | Expression treatment |
| 9 | Expression treatment |
| 10 | Expression treatment |
| 11 | REX control |
| 12 | REX control |
| 13 | REX control |
| 14 | REX treatment |
| 15 | REX treatment |
| 16 | REX treatment |

**Table S3.** Details of the LC-MS settings for the proteomic experiments

| **Parameters/instrument** | **HF** | **Exploris** | **Lumos** |
| --- | --- | --- | --- |
| **Gradient time (min)** | 90 | 90 | 95 |
| **MS scan range (m/z)** | 375-1500 | 375-1500 | 375-1500 |
| **HCD collision energy** | 33 | 33 | 35 |
| **Orbitrap resolution** | 120,000 | 120,000 | 120,000 |
| **MS^2^ resolution** | 45,000 | 45,000 | 60,000 |
| **MS AGC target** | 3e6 | 3e6 | 2.5e5 (250%) |
| **MS^2^ AGC target** | 2e5 | 2e5 | 2.5e5 |
| **MS maximum injection time (ms)** | 100 | 50 | Auto |
| **MS^2^ maximum injection time**  **(ms)** | 120 | 120 | Auto |
| **Isolation window** | 1.6 | 1.6 | 1.6 |
